# Therapeutic perspectives of exosomes in glucocorticoid-induced osteoarthrosis

**DOI:** 10.3389/fsurg.2022.836367

**Published:** 2022-08-02

**Authors:** Bin Lv, Zhangrong Cheng, Yajie Yu, Yuhang Chen, Weikang Gan, Shuai Li, Kangcheng Zhao, Cao Yang, Yukun Zhang

**Affiliations:** Department of Orthopaedics, Union Hospital, Tongji Medical College, Huazhong University of Science and Technology, Wuhan, China

**Keywords:** Exosomes, glucocorticoid, osteonecrosis, femoral head, treatment

## Abstract

Exosomes are widely involved in a variety of physiological and pathological processes. These important roles are also hidden in the physiological processes related to bone. Chondrocytes, osteoblasts, synovial fibroblasts, and bone marrow mesenchymal stem cells produce and secrete exosomes, thereby affecting the biology process of target cells. Furthermore, in the primary pathogenesis of osteoarthrosis induced by steroid hormones, mainly involve glucocorticoid (GC), the exosomes have also widely participated. Therefore, exosomes may also play an important role in glucocorticoid-induced osteoarthrosis and serve as a promising treatment for early intervention of osteoarthrosis in addition to playing a regulatory role in malignant tumors. This review summarizes the previous results on this direction, systematically combs the role and therapeutic potential of exosomes in GC-induced osteoarthrosis, discusses the potential role of exosomes in the treatment and prevention of GC-induced osteoarthrosis, and reveals the current challenges we confronted.

## Introduction

Glucocorticoids are a class of steroid hormones that play an important role in regulating the body's development, growth, metabolism and immune function, and are also the most widely used and effective anti-inflammatory and immunosuppressive agents in clinical practice. However, long-term use of glucocorticoids can induce osteocyte apoptosis, sustained bone destruction, injury and apoptosis of bone microvascular endothelial cells (BMECs) in the femoral head, and inhibit angiogenesis accompanied by microcirculation disorders (1, 2). Therefore, long-term use of glucocorticoids can lead to Glucocorticoid-induced osteoporosis (GIOP), Osteonecrosis of the femoral head (ONFH) and other osteoarthrosis.

GIOP (Glucocorticoid osteoporosis) is one of the most common and serious adverse reactions associated with glucocorticoid use, as considered to be the most common iatrogenic cause of secondary osteoporosis, leading to early and progressive bone loss, causing osteoarthritis pain and even pathological fractures, with postmenopausal women and men over 50 years of age at high risk (3). GC mainly acts directly on osteoblasts, osteoclasts and osteocytes. GC can reduce the formation of osteoblasts, promote the apoptosis of osteoblasts and osteoblasts, and prolong the life span of osteoclasts, means inhibit bone formation and promote bone resorption. It also reduces vascular endothelial growth factor bone vessels, interstitial fluid, and bone strength (4). At present, the clinical treatment of GIOP mainly includes the combined use of calcium and vitamin D, and the treatment of anti-osteoporosis drugs, including bone resorption inhibitors: bisphosphonates, sex hormone replacement therapy and thyrocalcitonins, when necessary. And bone formation promotors: parathyroid hormone amino-terminal fragment (PTHl-34), fluorine preparations, etc. But long-term use of these drugs can also lead to adverse reactions such as gastrointestinal reactions, osteonecrosis of the jaw, musculoskeletal pain, elevated blood pressure, and kidney stones (3, 5). Therefore, we need new effective drugs to treat GIOP. ONFH (Osteonecrosis of the femoral head) is a disease of mesenchymal cells or bone cells characterized by impaired subchondral microcirculation, bone necrosis, and accumulation of microfractures (6, 7). As a disabling and progressive disease, ONFH is caused by the destruction or interruption of the blood circulation of the femoral head at the initial stage, followed by cell necrosis, which eventually leads to hip joint dysfunction (8, 9). The pathogenic factors of ONFH mainly involve traumatic (such as femoral neck fracture, hip dislocation) and non-traumatic (such as corticosteroids, alcoholism, coagulopathy) risk factors (7, 10–12). As the most common type of ONFH, steroid-induced osteonecrosis of the femoral head (SONFH) accounts for 46.03% of the 15,000–20,000 new ONFH cases in China each year (6, 12). If early intervention is not provided, about 80% of patients will develop femoral head collapse, hip joint dysfunction, and permanent disability (13). The exact mechanism of GC (glucocorticoid)-induced ONFH involves cell death, vascular damage, or insufficient bone repair (14, 15). As we all know, GC directly induces apoptosis and inhibits angiogenesis, so it plays a vital role in destroying bone tissue formation and the occurrence of ONFH (14, 16–23). ONFH is a chronic disease that seriously affects the life quality of patients. ONFH, which usually occurs in young patients, may cause the femoral head to collapse and even require the replacement of all hip joints, accompanied by systemic functional defects and serious defects (24–26). So far, a variety of surgical methods have been used for total hip replacement and autologous cell transplantation. However, no treatment can completely cure the disease (27, 28). Besides, non-surgical treatments for ONFH (such as acetaminophen and cortisone injection) are not sufficient to prevent joint damage, and traditional drugs cannot restore the normal structure and function of the damaged musculoskeletal system (29). Therefore, it is imperative to explore the pathogenesis of ONFH in depth and find a new type of treatment that contribute to delaying the progression of the disease and repairing the damage of the bone marrow microenvironment. Luckily, the discovery of exosomes may have great potential and multiple advantages in the pathogenesis, prevention, and treatment of GC-induced osteoarthrosis (30–33).

Exosomes are naturally derived 50–150 nm nanocapsules that are secreted by cells and commonly found in blood, urine, saliva, cerebrospinal fluid, pleural fluid, and milk. Exosomes play an important paracrine effector role in cell-to-cell and/or cell-to-tissue communication and cross-species communication by transferring proteins and genetic material to target cells (34, 35). Exosomes usually contain various biologically active molecules, such as protein, RNA (mRNA, microRNA, and other non-coding RNA), DNA (mitochondrial DNA [mtDNA], double-stranded DNA [dsDNA], single-stranded DNA, and viral DNA), Lipids, amino acids, and metabolites. These different components play a key role in signal transduction between cells and regulate the microenvironment of nearby or distant cells (36–38). As a new type of biological vesicles, Exosomes have multiple advantages and are considered to be suitable tools for the treatment of various diseases including cancer. First of all, most cells can secrete exosomes and retain the characteristics of parental cells. Secondly, unlike a single protein or small molecule, exosomes contain molecules with heterogeneous functions but lack the complexity of cells and organs. In addition, exosomes show many benefits in terms of biocompatibility, immunogenicity, stability, pharmacokinetics, biodistribution, and cellular uptake mechanisms. Bone-derived exosomes are believed to be essential for intercellular communication between bone cells. The exosome-mediated transfer of nucleic acid or protein cargo between bone cells can bypass the space barriers between different cells and play a vital role in the crosstalk between bone cells that regulate bone homeostasis. Since exosomes are a new biological vesicle that regulates the bone formation, we summarized the characteristics of exosomes, listed the known functions of exosomes in bone homeostasis, and discussed the clinical potential.

In this article, we will mainly through the example of GC-induced GIOP and ONFH introduce the mechanism of exosomes in GC-induced osteoarthrosis and describes the latest achievements in the treatment of GC-induced osteoarthrosis by exosomes. Then, we introduced how exosomes act on GC-induced osteoarthrosis in different aspects. Finally, we discussed the problems that must be solved in the clinical application of these methods and the future research direction of exosomes in the treatment of GC-induced osteoarthrosis.

**Figure 1:**
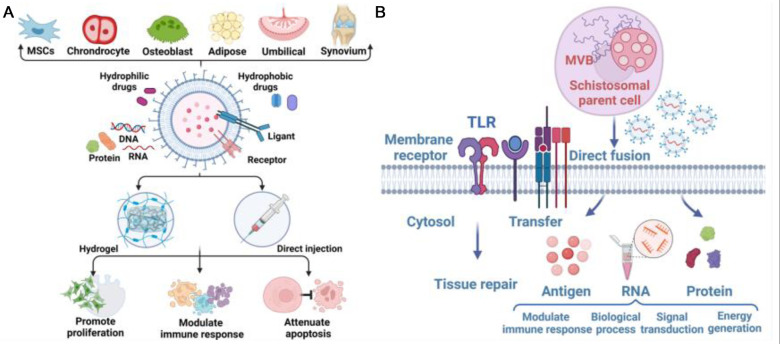
(**A**) Exosome-deriving cells and application of exosome to defect sites, achieving promotion of cell proliferation, modulation of immune response, and attenuation of apoptosis. (**B**) Biology and functions of exosomes in Glucocorticoid-induced Osteonecrosis of the Femoral Head (ONFH). Exosomes can be regarded as vehicles for delivering antigens, proteins and RNA to modulate immune responses, gene expression, and metabolic processes. Exosomes are also involved in the transfer of lipids to recognize TLRs, thus participating in tissue repair.

As a new and potential substance that can be used for early intervention and treatment of GC-induced osteoarthrosis, exosomes have been found to play an important role in the pathogenesis of osteoarthrosis caused by GC. We summarized the roles of exosomes in the three main mechanisms of GC-induced osteoarthrosis (Figure 1).

### The role of exosomes in GC-induced apoptosis

Studies have shown that GC can directly act on osteoblasts, osteoclasts and osteocytes, reduce the formation of osteoblasts and promote the apoptosis of osteoblasts and osteoblasts. For osteoblasts, activation of glucocorticoid receptors up-regulates the expression level of P53 in mouse osteoblast cell line Mc3t3-e1, thereby enhancing the transcriptional activity of P53 and leading to up-regulation of pro-apoptotic genes P21, PUMA and NOXA. Finally, Mc3t3-e1 cells were induced apoptosis and cell cycle arrest (39). Deng's study found that dexamethasone can down-regulate the expression of P-PI3K and P-Akt to inhibit the activation of PI3K/AKT signaling pathway. The expression of Bax, caspase3, caspase9 and bcl-2 could be decreased and the expression of Bcl-2 could be increased to reduce dexamethasone induced osteoblast apoptosis by removing the expression of GSK3β, the downstream target of PI3K/AKT (40). In addition, GCs’ dose has different effects on bone cells. Low dose GC treatment can lead to autophagy of bone cells, while under high dose GC stress, bone cells may undergo apoptosis or necrosis (41).

Accumulated studies have shown that GC leads to the occurrence and development of ONFH through a variety of mechanisms, and GC-induced bone cell apoptosis is one of the most important ways (42, 43). Under the action of GC, a large number of bone cells undergo apoptosis, leading to loss of bone strength, and disease progression eventually leads to the collapse of the femoral head (8). The research of Hamamura and Saito et al. showed that the increase of bone cell apoptosis is related to endoplasmic reticulum (ER) stress. Specifically, the accumulation of misfolded or unfolded proteins induces phosphorylation of protein kinase-like endoplasmic reticulum kinase (PERK), activates the unfolded protein response (UPR), and helps cells adapt to ER under mild ER stress conditions Stress (44). Among the three main signal pathways of ER stress, the PERK (protein kinase RNA-like ER kinase)/CHOP (CCAATenhancer-binding protein homologous protein) pathway is considered to be closely related to apoptosis. CHOP can inhibit the expression of Bcl-2, increase the level of lytic caspase-3, and cause cell apoptosis.

**Figure 2:**
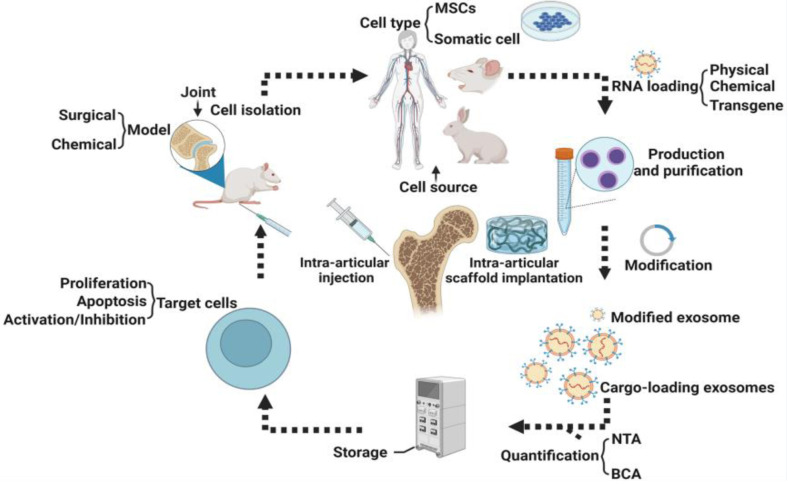
Procedures for preparation of exosomes.

More and more studies have shown that exosomes play a crucial physiological and pathological role by influencing cell apoptosis, and the same role also occurs in bone physiology (45, 46). For example, exosomes from human umbilical cord mesenchymal stem cells (HUCMSC) can reduce apoptosis of bone marrow mesenchymal stem cells (BMSC) in osteoporotic rats through Mir-1263/Mob1/Hippo signaling pathway (47). In another study, EXOs derived from adipose-derived MSCs (ADSCs-EXOs) prominently reduced H/SD (hypoxia and serum deprivation)-induced apoptosis in the osteocyte-like cell line MLO-Y4 cells by increasing the ratio of Bcl-2/Bax, reducing the production of reactive oxygen species and cytochrome c, and activating caspase-9 and caspase-3 subsequently (48). A research report from S3 shows that exosomes (PRP-EXOS) derived from PRP (Platelet-rich plasma) promote the expression of BCL-2 under ER stress through the AKT/BAD/BCL-2 signaling pathway. The ability to prevent GC-induced apoptosis in ONFH rats (49). PRP-exos significantly enhances the activation of the AKT and ERK signaling cascade, on the one hand, it promotes the angiogenesis of the bone microenvironment; on the other hand, it promotes the expression of anti-apoptotic proteins such as bcl-2 (50). Secondly, the study of S. Zhang et al. observed that exosomes were activated by CD73-mediated AKT and ERK signals to increase the expression of chondrocyte proliferation and anti-apoptotic related genes (51). Guo et al. discovered for the first time that exosomes secreted by human synovial-derived mesenchymal stem cells (SMSC-Exos) can be internalized into bone marrow-derived stromal cells (BMSCs) and enhance their proliferation and anti-apoptotic ability. In in vivo experiments, they found that infusion of SMSC-Exos can reduce GC-induced trabecular bone loss, bone marrow necrosis, and fat cell accumulation. The infusion of SMSC-Exos can effectively prevent GC-induced ONFH in the rat model. At the same time, in vitro cell experiments also found that SMSC-Exos can reverse the anti-proliferative effect induced by GC (52). According to reports, SMMSC-Exos derived from SMMSC significantly reduced glucocorticoid (GC)-induced adipocyte aggregation, bone marrow necrosis, and trabecular bone loss, and to a certain extent reversed bone cell proliferation arrest and BMSC cells apoptosis (52). Micro-CT analysis also showed that SMMSC-Exos significantly improved the trabecular bone microstructure and mineral density of ONFH (ONFH) induced by GC in rats (52).In addition, exosomes produced by MSC can prevent bone cell apoptosis in hypoxia/serum deficiency models and glucocorticoid-induced osteonecrosis models (48, 53) (Figure 2).

The numerous pieces of evidence indicate that exosomes from various sources can inhibit GC-induced apoptosis of osteoblasts and bone cells, and reverse osteonecrosis to a certain extent, which shows the great potential of exosomes in the early treatment of osteoarthrosis caused by GC.

### The role of exosomes in GC-induced vascular damage

In the pathogenesis of osteoarthrosis caused by GC, GC damage to vascular endothelial cells is another main way. A considerable number of studies have shown that: glucocorticoids can induce injury and apoptosis of bone microvascular endothelial cells (BMECs) of the femoral head, which is closely related to the development of osteonecrosis and osteoporosis, leading to a hypercoagulable state and abnormal microthrombosis in the area of ONFH, and severely reducing the blood supply of trabecular bone (22, 54–58). The work of Greenberger et al. 2010 found that: GC treatment can inhibit the expression of VEGF-a in ECS and subsequent angiogenesis (59). Vascular damage is manifested by decreased function of circulating angiogenic cells, decreased migration function, and VEGF protein secretion (60).

Therefore, inhibition of endothelial cell (EC) apoptosis is necessary to maintain the integrity of blood vessels and prevent the further development of GC-induced osteoarthrosis (61). Similarly, exosomes also play an important role in the physiological and pathological processes related to endothelial cells. The study by Hu GW et al. pointed out that exosomes secreted by mesenchymal stem cells derived from human induced pluripotent stem cells can reduce limb ischemia by promoting angiogenesis in mice (62). Anderson et al. reported that MSC-derived exosomes contained abundant angiogenesis-related proteins that promote endothelial cell proliferation and angiogenesis (63, 64). And the team also detected the promotion of angiogenesis and tissue repair (including bone formation) by exosomes in both in vivo and in vitro experiments (65).In an animal experiment with osteoporotic rats as the experimental object, eight weeks after implantation of MSC-derived exosomes, the experimental group of rats detected the strong formation of blood vessels and bone tissue that was not in the control group (62). In addition, Yokota et al. proved that exosomes can accelerate the surgical angiogenesis of vascular implantation into the necrotic bone by injecting PRP-containing exosomes (66). In addition, activated platelets can also promote the proliferation and migration of bone mesenchymal stem cells (BMSCs) and ECS, thereby promoting bone formation and capillary formation (67). Qi et al and other studies have also shown that in ovariectomized rat models, exosomes secreted by iPS-derived MSCs can promote the regeneration of bone defects by enhancing angiogenesis and bone formation (68). Zuo et al. used miR-26a transfected human CD34 + stem cell-derived exosomes and found that miR-26a-CD34+-exosome enhanced the ability of human umbilical vein endothelial cells to migrate and form blood vessels, indicating that this kind of exosomes can prevent glucocorticoid-induced necrosis of the femoral skull by promoting angiogenesis and osteogenesis (69). These findings provide a novel method for vascular remodeling and bone cell proliferation in soft tissues to enhance early tissue repair.

### The role of exosomes in GC-induced insufficient bone repair

The pathogenesis of ONFH caused by GC is in addition to inducing osteoblast apoptosis, damage to vascular endothelial cells also involves its inhibition of bone formation (70–73). Previous research reports pointed out that GC has complex stimulating and inhibiting effects on bone metabolism. During normal bone formation, an appropriate amount of endogenous GC signal is necessary. For example, a study by Phillips JE showed that a small dose of dexamethasone (Dex) can promote the differentiation of several osteoblasts in the oval system. However, the use of high-dose GC significantly reduced the patient’s bone mass and lowered bone density, which ultimately greatly promoted the occurrence and development of ONFH (73–75). Similarly, another study also showed that GCs down-regulated the expression of osteogenic marker molecules Runt-related transcription factor 2 (RUNX2) and alkaline phosphatase, and was associated with the decrease of bone density and the rupture of trabecular bone (75). Interestingly, the findings of Ekstrom et al. found that fusion of monocyte-derived exosomes with MSC can trigger the up-regulation of two osteogenic markers: RUNX2 and BMP-2. This phenomenon and two other studies both show that exosomes can interact directly with bone cells, thereby affecting the process of bone formation (76, 77). Of course, the evidence that exosomes play an important role in osteogenesis is not limited to this limited study. As we all know, bone remodeling is a complex process that mainly involves two steps: osteoclastogenesis (used to remove damaged bone tissue) and osteogenesis (used for bone formation). Current research reports have shown that exosomes play an important role in these two steps. Published reports indicate that the transfer of exosome-specific proteins, mRNA, and miRNA is the main mechanism of exosome-mediated bone remodeling. This crosstalk establishes a new network of cell-cell interactions during bone homeostasis (78). For example, the study of Cui, Y, and her colleagues found that mature osteoblast-derived exosomes may trigger the mutation of miRNA expression profile, and then cooperatively inhibit the expression of Axin1, the core component of the Wnt signaling pathway, and finally, β-catenin is up-regulation, leading to enhanced osteogenic differentiation (79). Besides, Let-7-rich exosomes derived from osteoblasts can also enhance osteogenic effects by regulating AT-hook 2 (HMGA2) and AXIN2 (79, 80). The proliferation induced by MSC-derived exosomes has also been reported, and the MAPK pathway may be a key factor in the activity of osteoblasts mediated by exosomes (81).In addition to physiological conditions, exosomes also exhibit important functions related to osteogenesis under pathological conditions. The research results of Furuta et al. showed that during fracture healing, exosomes derived from bone marrow stem cells express MCP-1, MCP-3, SDF-1, angiogenic factors, mRNA, and miRNA, and jointly promote bone Reshape (82).At the same time, exosomes may also increase osteoblast-related proteins (RUNX-2, ALP, OCN, and OPN) and some genes (miRNA-196a, miRNA-27a, and miRNA-206) to enhance the proliferation and differentiation of osteoblasts (83). In addition to the participation of osteoblasts in bone remodeling, osteoclasts also play an important role, and the proper balance between the two is the key to complete thigh remodeling. Exosomes also play an important role in mutual signal communication between osteoblasts and osteoclasts. The inactivation of the RANK-RANKL signaling pathway in osteoblasts can release exosomes containing miR-503-3p, thereby inhibiting the formation of osteoclasts. In animal experiments, in the CD9−/− mouse femoral fracture model (in which the production of exosomes was inhibited), the formation of callus in the experimental group was significantly delayed compared with the control group. However, this delayed effect can be corrected by local injection of exosomes (82).Similar functions of exosomes in promoting fracture repair and bone remodeling have also been verified in a mouse model of osteoporosis (62). This series of research results suggest that exosomes may play an irreplaceable role in the process of bone remodeling and bone repair, and these phenomena may be occurring in the bone repair process of ONFH caused by GC. This hypothesis has also been confirmed by research by Zuo and his colleagues. Their experiments suggested that miR-26a-CD34+-exosome enhanced the osteogenic differentiation of BMSCs under the influence of GC. Finally, miR-26a-CD34+-exos increased the vascular density and small bone density of the femoral head in the GC-induced ONFH mouse model, thereby inhibiting the progression of ONFH and promoting bone repair (69). Shang-Chun Guo et al. also found that SMSC-Exos can improve bone mineral density and trabecular bone microstructure of GC-induced ONFH rats. Immunohistochemical staining for osteocalcin showed that MPS (methylprednisolone) was injected into the thigh The osteogenic response of bones is reduced, but SMSC-EXOS significantly inhibits this effect (52). Another study found that exosomes rich in miR-122-5 down-regulate SPRY2 through the RTK/Ras/mitogen-activated protein kinase (MAPK) signaling pathway, thereby delaying the development of ONFH (84).

In the past few decades, exosomes are involved in many biological processes related to bone metabolism, including angiogenesis, cell differentiation, immune regulation, metabolic balance, and development (36, 85–88). However, exosomes are not simple nucleic acid or protein molecules, but microvesicles containing a variety of substances including RNA, DNA, protein, and lipids. While there are extensive biological functions, exosomes are also highly heterogeneous, involving different sources and different contents. Therefore, since exosomes work through each of the molecules contained, understanding the mechanism of action of each content is crucial for further understanding and application of exosomes. We reviewed the roles played by different contents of exosomes derived from cells related to bone metabolism and the molecular mechanisms of their effects and summarized the possible roles of various substances in GC-induced osteoarthrosis (Table 1).

**Table 1:** Function of RNA family in glucocorticoid-induced osteonecrosis of the femoral head.

Class	Molecule	Vitro study	Vivo study	Biological effects	Ref.
miRNA	miR-26a	BMSCs	GC-induced ONFH rats	promoted the osteogenic differentiation of BMSCs, increased the vessel density and trabecular bone integrity in the GC-induced ONFH	(69)
	miR-548d-5p	hBMSCs	/	suppressed the dexamethasone-induced adipogenic differentiation of hBMSCs, enhanced their osteogenic potential.	(99)
	miR-27a-3p	MC3T3-E1 cells	/	decreased adenomatous polyposis coli (APC) expression, activated β-catenin pathway	(89)
	miR-8485	BMSCs	/	activated Wnt/b-catenin pathways, promoted chondrogenic differentiation of BMSCs	(92)
	miRNA-122-5p	BMSCs	ONFH rabbits	down-regulated SPRY2, promoted the proliferation and differentiation of osteoblasts, attenuated ONFH development	(153)
IncRNA	lncRNA-KLF3-AS1	OA chondrocytes	OA mice	induced chondrocyte proliferation, inhibited chondrocyte apoptosis via miR-206/GIT1 axis.	(109)
	lncRNA HOTAIR	MSCs	/	Regulated osteogenic differentiation and proliferation, targeted gene SMAD7 in non-traumatic ONFH	(110)
	lncRNA-Miat	rMSCs	/	promoted osteogenesis of rMSCs while silencing, modulated the function of endothelial cells via VEGF	(114)
circRNA	circUSP45	BMSCs	SD rats	sponged miR-127-5p through PTEN/ AKT signal pathway, reduced osteogenesis in bone GIONFH	(126)
	circ19142/circ5846	MC3T3-E1 cells	/	induced osteogenic differentiation	(127)
	circFOXP1	MSCs	Wistar rats	promoted proliferation and differentiation of MSCs, preserved the MSC multipotent state, modulated non-canonical Wnt and EGFR pathways	(128)
	circRNA0010729	HUVECs	/	regulated hypoxia-induced HUVECs via miR-186/HIF-1a axis	(130)
	circRNA0003575	HUVECs	/	promoted the proliferation and the angiogenesis ability of oxLDL-induced HUVECs while silencing	(131)

### The role of miRNA

As one of the most studied contents in exosomes, miRNA released by exosomes has been shown to play an important role in multiple physiological processes of bone metabolism. For example, exosomes derived from myoblasts enter pre-osteogenic cells and promote osteoblast differentiation through miR-27a-3p-mediated β-catenin pathway activation (89). Young MSC exosomes can rejuvenate senescent HSCs through autophagy-related miR-17 and miR-34a cell-to-cell transfer, while miR-23b and miR-92a can effectively treat OA (Osteoarthritis) (90, 91). Furthermore, the exosomes of chondrocytes may promote the chondrogenesis and differentiation of BMSCs by activating the Wnt/β-catenin pathway, which is related to the inhibition of GSK-3β expression by miR-8485 in the exosomes (92).

Besides, recent studies have also highlighted the importance and significance of microRNA (miRNA) in the pathogenesis, prevention, and treatment of GC-induced osteoarthrosis (31, 32). One study showed that exosomal miRNAs promote osteoarthrosis development by influencing osteoblasts, osteoclasts and bone matrix through oxidative stress (OS) mediation. Exogenous antioxidants can help prevent or delay the development of osteoarthrosis, while the antioxidant balance in the body is disrupted (93). But Chen et al. detected the expression of Mir-425-5p in bone marrow mesenchymal stem cells (MSC) by quantitative reverse transcriptase-polymerase chain reaction (qRT-PCR) and the expression of TNF by ELISA, and the results showed that Mir-425-5p could regulate cell apoptosis, proliferation and differentiation induced by TNF. ANXA2 is a target of Mir-425-5p and is involved in TNF-induced apoptosis, proliferation and differentiation of MSC cells. It was concluded that Mir-425-5P could enhance osteoporosis in mice (94). The above studies indicate that the mechanism of miRNA action on osteoporosis still needs further study. The above study indicates that the current research on the mechanism of miRNA action on osteoporosis is limited, and it is necessary to conduct in-depth basic and clinical research.

Wu et al. verified three up-regulated miRNAs (miR-210-3p, miR-320e, and let-7c) by comparing the expression of miRNA in non-traumatic ONFH and femoral neck fractures (95).In previous research evidence, Let-7 in osteoblast-derived exosomes has been shown to enhance osteogenesis by regulating AT-hook 2 (HMGA2) and AXIN2 (79, 80). This indicates that there are still a large number of miRNA that may have a potentially important role in bone repair and bone remodeling in ONFH, waiting to be discovered and explained. ONFH caused by overuse of glucocorticoids accounts for the majority of non-traumatic ONFH. The decrease in the proliferation of mesenchymal stem cells is related to the pathogenesis of glucocorticoid-induced ONFH, and this mutual connection may be involved in the exosomes released by mesenchymal stem cells. Bian et al. compared the expression of miRNA in human mesenchymal stem cells treated with and without dexamethasone. The study found that 11 up-regulated (miR-16-5p, miR-103a-3p, miR-107, miR-196a/b-5p, miR-378d, miR-1268a/b/f/g, miR-4289) and 6 down-regulated (miR-24-3p, miR-378a/h/I, miR-4448, miR-4634) miRNA were found between the two different concentrations of dexamethasone treatment groups. For further analysis, they injected methylprednisolone (21 mg/kg) subcutaneously into C57BL/6J mice and found that miR-21-3p and miR-652-5p were up-regulated and miR-34b-3p, miR-34c-5p, miR-148a-3p, miR-196a-5p, and miR-206-3p are down-regulated, which are predicted to be involved in osteogenic differentiation (96). Hao et al. found that miR-708 may enhance the osteogenic effect of mesenchymal stem cells and inhibit their adipogenic differentiation ability by targeting Smad3 (8). Yamasaki et al. confirmed that miR-210 (angiogenic miRNA) is highly expressed in non-invasive ONFH and may regulate angiogenesis in ONFH (56, 97, 98). Sun et al. confirmed that miR-548d-5p promotes the osteogenic differentiation of mesenchymal stem cells by acting on PPARγ, and may inhibit glucocorticoid-induced ONFH (99). In addition, miR-27 has also been shown to inhibit adipogenesis and enhance bone formation by regulating the expression of GREM1 and PPARγ (83, 100–103). These findings indicate that miRNAs secreted in the bone marrow microenvironment play an irreplaceable role in the pathogenesis of steroid-induced ONFH and the balance between osteogenic differentiation and adipogenic differentiation of mesenchymal stem cells.

### The role of lncRNA

As a regulatory RNA, long non-coding RNA (lncRNA) has been shown to play a key role in various cellular physiological functions including cell proliferation, invasion, metabolism, apoptosis, and stem cell differentiation. Recent studies have shown that lncRNA is directly involved in the pathogenesis of many orthopedic diseases and also plays an important role in the process of bone development and regeneration. For example, long non-coding RNA (lncRNA) has been shown to be an important exosomal content in OA, widely involved in the regulation of various pathological and physiological processes (103, 104). Exosomes from adipose-derived stem cells (ADSCs-EXOS) have been verified that play an effective part in the repair of different tissues and organs. ADSCs-EXOS have also been confirmed to help in the treatment of osteoporosis (105). However, Wang et al. believed that compared with ADSC-EXOS, KCNQ1OT1-ExOS, as a kind of lncRNA closely related to cell proliferation, migration and apoptosis, had a more significant inhibitory effect on TNF-α -induced cytotoxicity and apoptosis (106).

Recent research results indicate that lncRNA also plays an important regulatory role in the pathogenesis and repair of ONFH. LncRNA was found to be differentially expressed in ONFH tissues, bone marrow mesenchymal stem cells and bone microvascular endothelial cells which isolated from ONFH patients (9, 107, 108). Functional research has further clarified its important role in the survival of osteoblasts closely related to ONFH and the osteogenic differentiation of bone marrow mesenchymal stem cells. Liu et al. reported that MSC-Exos mainly up-regulated Col2a1 and proteoglycan levels through lncRNA-KLF3-AS1.239 released from exosomes, and down-regulated the expression of MMP13 and Rux2, which promoted the survival of IL-1β-treated chondrocytes (109). According to reports, as a differentially expressed lncRNA isolated from steroid-induced ONFH patients, forced expression of RP11-154D6 can promote the increase in the expression of osteogenic differentiation markers (osteocalcin (OCN) and RUNX2) and reduce the expression of adipogenic differentiation markers (such as lipoprotein lipase (LPL) and peroxisome proliferator-activated receptor gamma (PPAR gamma)), these effects ultimately lead to enhanced bone formation (108). In another study, Wei et al. found that HOTAIR can negatively regulate the proliferation and osteogenic differentiation of mesenchymal stem cells by regulating the expression of miR-17-5p and Smad7, and can be used as a therapeutic target for non-invasive ONFH (110). Wang et al. used the reconstruction of the coding-noncoding gene co-expression (CNC) network to reveal the key role of two lncRNAs (HOTAIR and RP1-193H18.2) in regulating the osteogenic and adipogenic differentiation of bone marrow MSCs (111). In addition, Yu et al. analyzed the BMEC (bone microvascular endothelial cells) of patients who underwent a conventional total hip replacement and exposed the cells to hydrocortisone (0.1 mg/ml) for 24 h using the co-expression analysis technology of non-coding RNA and related mRNA, the results reveal that FoxO transcription factors are closely related to the regulation of angiogenesis (112, 113). Furthermore, the overexpression of MIAT in the bone marrow microenvironment may lead to steroid-related ONFH by inhibiting the osteogenic differentiation of MSC, and this process can be blocked by the epigenetic silencing of MIAT by HXTL (114). Fan and colleagues confirmed that MALAT1 can protect human osteoblasts from dexamethasone-induced cell death. Specifically, MALAT1 prevents steroid-induced ONFH by regulating PPM1E-AMPK-NRF2-oxidative stress and the miR-214-ATF4 axis (32).

### The role of circRNA

Circular RNA (circRNA) is a member of the non-coding RNA family. Unlike linear RNAs such as miRNA or lncRNA, it forms a covalently closed continuous loop, making them resistant to digestion by RNA exonuclease. Accumulated evidence shows that circRNA can perform biological functions by acting as a microRNA sponge, encoding proteins, and binding to proteins (115–121). Research on circRNA is later than most linear RNAs, but in recent studies, circRNA has also been found to be involved in bone metabolism in many diseases (including GC-induced osteoarthrosis).

For instance, Feng et al. found that hsa_circ_0006859 in exosomes of osteoporosis patients can inhibit osteoblast differentiation and promote adipose decomposition of human bone marrow mesenchymal stem cells (hBMSCs). Hsa_circ_0006859 acts, as a competitive endogenous RNA (ceRNA) of Mir-431-5p, directly binds to Mir-431-5p and promotes the expression of ROCK1 which was confirmed as a novel target gene of Mir-431-5p (122).

Generally, the weakened osteogenic differentiation and increased adipogenic differentiation of BMSCs are closely related to the formation of ONFH (102). Xiang et al. have identified 90 up-regulated and 141 down-regulated differentially expressed circRNAs in steroid-induced ONFH (SONFH) BMSCs (123). Further functional studies have found that circRNA immunoglobulin superfamily member 11 can promote osteoblast differentiation in BMSC osteogenesis through glycogen synthase kinase 3β/β-catenin signaling pathway, and knocking down this circRNA can increase miR199b-5p expression (123–125). In addition, some studies have found that circRNA plays a key role in the regulation of bone metabolism mainly by acting as a molecular sponge of miRNA. For example, Kuang et al. proved that in the steroid-induced ONFH rat model, circRNA ubiquitin-specific protease 45 can upregulate phosphatase and tensin homologs by binding to miR-127-5p, thereby inhibiting the protein kinase B pathway and regulate the bone mass of rats (126). In addition, the mode of action of the miRNA-mRNA axis targeted by circ19142/circ5846, circ19142 and circ5846 have been shown to act as sponges for miR-7067-5p in osteoblast differentiation (127). Besides, circRNA FOXP1 has also been shown to play a key role by acting as a sponge for several miRNAs in the regulation of MSC differentiation, which is closely related to the pathogenesis of ONFH (128). Although there are few studies on another important pathogenic mechanism (adipogenic differentiation of mesenchymal stem cells) that affects osteogenesis, The above observation results also show that there is a close correlation between circRNA and SONFH, which can be used for follow-up research and clinical treatment, and provides a good guide for finding therapeutic targets. In another core pathogenic mechanism of ONFH, endothelial cell damage and angiogenesis disorder, circRNA has also been shown to play an important biological role (129). For example, CircRNA0010729 mediates the apoptosis and proliferation of vascular endothelial cells by targeting the miR-186/hypoxia-inducible factor-1α axis (130). Furthermore, circRNA0003575 is up-regulated in human umbilical vein endothelial cells (HUVEC) induced by oxidized low-density lipoprotein and promotes HUVEC proliferation and angiogenesis (131). Although there is no research on circRNA directly targeting endothelial cells in the bone marrow microenvironment, these findings also indicate that circRNA may play an important role in the activation mechanism of ONFH. The above research results indicate that circRNA plays a unique role in the formation of ONFH, and due to its unique stability, may play an irreplaceable role in the treatment of ONFH.

### The role of protein

As a kind of exosomal load, many types of specific cell proteins have been shown to contribute to the communication and signal transduction between cells (132–137). In the study of bone-related exosomal proteins, Tsuno et al. used 2D-DIGE and mass spectrometry to identify serum exosomal proteins extracted from the healthy group and the OA group. They found that the exosome between the OA group and the healthy group has 21 spots in the somatic protein profile with different intensities, such as cathepsin F and Igalpha-2 chain C region, indicating the potential role of these proteins in OA (138). At the same time, recent studies have also discovered the role of exosomal proteins in regulating the biological response of chondrocytes. Zhang et al. found the expression of CD73/ecto-5'-nucleotidase in MSC-derived exosomes and found that the CD73 inhibitor AMPCP or the non-selective adenosine receptor antagonist theophylline can reduce MSC Exosomes-induced phosphorylation of AKT and ERK in chondrocytes (139). The results above indicate that the role of the protein-loaded exosomes in the differentiation and development of bone cells still needs further exploration, although the existing evidence has suggested its regulation of cartilage and MSC.

### The role of DNA

Since the study of exosomal DNA is later than the study of RNA, only a small amount of literature has reported that carrying cytoplasmic DNA in exosomes can prevent cell senescence and cell death caused by DNA damage (140, 141). Moreover, exosomal DNA can exert effects because cells can secrete exosomes and remove harmful DNA in the extracellular matrix. In addition to double-stranded DNA, exosomes also contain single-stranded DNA, but we still know little about the biological role of this DNA. Therefore, it is necessary to study the expression and function of these DNAs in the bone marrow microenvironment.

## Conclusions and outlooks

Exosomes carrying contents like DNA or RNA family serve as crucial vehicles for intercellular communication. Although there is a broad range of potential applications and uses of exosomes, it still appears to be some problems of methods for exosome isolation and analysis. Primarily, the quantities of exosomes released by mammalian cells is relatively low and the purification of exosomes is burdensome. Enhancing the ability to load a variety of cargoes and targeting capabilities without corrupting exosomes is also very important for the utility of this delivery technology. It is hoped that more researchers will participate in the exploration of these problems from bench to bedside in the future. Exosomes show important regulatory effects in different stages and different pathological mechanisms in osteoarthrosis caused by GC, which shows the usefulness and potential of exosomes in the treatment of steroid-related osteoarthrosis. Up to now, take GC-induced ONFH’s treatment for example, it has mainly relied on drug therapy, core decompression, interventional therapy, and cell therapy as early intervention methods, but usually, 65%–85% of patients will continue to develop femoral head collapse (85). Once it develops into the terminal stage of the disease, total hip replacement surgery becomes the only viable option, and this will bring tremendous pressure on the patient's economy and life. Furthermore, for those young patients, ONFH often means that multiple revision surgeries may be required in the future (because the life of the prosthesis is limited), which aggravates the patient's physical and psychological burden. Therefore, as a promising alternative to the traditional treatment of osteoarthrosis, exosomes have many incomparable advantages in the early intervention of osteoarthrosis, and they have received widespread attention as a new treatment for osteoarthrosis (34, 142, 143). Firstly, exosomes have multiple advantages in immunogenicity, and allogeneic exosome injection may not cause obvious complications and rejection in terms of immunogenicity (66). Secondly, exons show good stability and pertinence, because they maintain the properties of their parent cells for a long period and maintain their inherent integrity, which makes them more effective in the treatment of osteoarthrosis. Easily target cells without causing systemic adverse reactions (144–146). Finally, exosomes also show certain advantages in biodistribution and pharmacokinetics. Due to their small size, these nanoparticles can easily reach the wound site. Exosomes can be transformed to express specific surface molecules and can selectively bind to molecules overexpressed on target cells, and exosomes can use their unique functions to extend their half-life (147–152). However, exosomes still face many challenges before entering clinical applications, and the main resistance comes from the separation and purification of exosomes, the modification of exosomes, and the heterogeneity of exosomes. Concerning the role of exosomes in GC-induced osteoarthrosis, research on the underlying mechanism and diagnostic/therapeutic applications have just begun. Although there are still many problems to be solved in this field, we speculate that technological advancement will give an optimistic outlook for the treatment of GC-induced osteoarthrosis based on exosomes.

## Data Availability

The original contributions presented in the study are included in the article/Supplementary Material, further inquiries can be directed to the corresponding author/s.
